# Patients with advanced pancreatic and biliary cancer appear vulnerable to SARS-CoV-2 Omicron variant: An observational study during the COVID-19 outbreak in Shanghai

**DOI:** 10.3389/fonc.2023.1115293

**Published:** 2023-03-22

**Authors:** Tao Han, Lujun Chen, Jia Gu, Shen Wu, Maiweilan Maihemuti, Jue Yang, Hao Wang, Jun Wu, Yue Zhang, Yun Cong, Jiening Wang, Tingsong Chen

**Affiliations:** ^1^ Department of Medical Oncology, The First Hospital of China Medical University, Shenyang, China; ^2^ The General Hospital of Northern Theater Command Training Base for Graduate, China Medical University, Shenyang, China; ^3^ Department of Otolaryngology, The First Affiliated Hospital of China Medical University, Shenyang, China; ^4^ Department of Oncology, Seventh People’s Hospital of Shanghai University of Traditional Chinese Medicine, Shanghai, China

**Keywords:** pancreatic and biliary cancer, COVID-19, SARS-CoV-2 omicron, immunosuppression, vulnerable population

## Abstract

**Background:**

The COVID-19 pandemic has spread rapidly across the globe. Cancer patients have a higher risk of severe infections and associated mortality than the general population. However, the lethal effect of Omicron-variant affection on advanced pancreatic and biliary cancer patients is still not clear. Herein, we designed an observational study to shed light on the influence of the Omicron variant on this so-called “King of Cancer” and improve management of these patients with COVID-19 in the future.

**Methods:**

Omicron-infected patients with advanced pancreatic and biliary cancer were enrolled from 15 April to 31 May 2022. Four groups were set up in this study: Group 1, Omicron-infected cancer patients (N = 4); Group 2, non-infected cancer patients (N = 4); Group 3, infected non-cancer-afflicted subjects (N = 4); Group 4, non-infected non-cancer-afflicted subjects (N = 4). On Days 0, 7, and 14 after infection, the blood samples were collected dynamically from all subjects. The primary endpoints were disease severity and survival.

**Results:**

At the endpoint of this observational study, Patient Nos. 2, 3, and 4 died separately on Days 11, 25, and 13 after viral infection. All of them had advanced cancer, with a death rate of up to 75%. Group 1 presented an overall T-cell exhaustion status compared with other groups. Group 1 had obviously lower T-cell populations and higher B-cell percentages and CD4^+^T/CD8^+^T ratios (P <0.05). Time-course cytokine monitoring results showed that IL-1β was significantly decreased in Group 1 (P <0.05) and generally kept at a low level without obvious fluctuation. IL-6 was markedly increased in infected cancer patients (P <0.01) but remained at a low level and had no apparent change during the whole infection process in non-cancer-afflicted subjects. Furthermore, several inflammatory parameter indexes indicated a tight association of Omicron infection with the disease course and prognosis of Omicron-infected cancer patients.

**Conclusions:**

Advanced pancreatic and biliary cancer patients with Omicron infection have severe symptoms and poor outcomes. More attention, protective measures, and routine healthcare services should be recommended to these vulnerable populations in clinical practice during the pandemic in the foreseeable future.

## 1 Introduction

The 2019 Coronavirus Disease (COVID-19) pandemic has had a tremendous impact on human life worldwide since 2019, resulting in over 6 million deaths globally at the time of writing (https://coronavirus.jhu.edu/map.html). In late February 2022, a wave of newly discovered severe acute respiratory syndrome coronavirus 2 (SARS-CoV-2) infections spread swiftly in Shanghai, China. As of 31 May 2022, 63,007 newly diagnosed cases and 595 death cases have been reported according to the Shanghai Municipal Health Commission (1). During the epidemic in Shanghai, the phylogenetic characteristics of the SARS-CoV-2 virus genomes in 129 cases were analyzed and compared with those in the GISAID database. The results showed that all the new viral genomes in Shanghai were SARS-CoV-2 BA.2.2 subspecies, which is a subline of the Omicron variety of SARS-CoV-2 (B.1.1.529) (1). Strict and comprehensive pandemic control strategies have been implemented immediately to reduce infected cases and buy time for full vaccination coverage during the epidemic in Shanghai in 2022 (1). Consequently, this rapid epidemic has had a large impact on the health care system. Most of the healthcare activities, such as chronic disease management, cancer diagnosis, and systemic treatments, have been delayed or cancelled during these periods, with increases in basic disease-related mortality predicted, especially for cancer patients with advanced disease.

Pancreatic and biliary cancers are malignant tumors with the worst prognosis (2, 3). In Shanghai, the prevalence rate of pancreatic and biliary cancer is increasing year by year, and that of biliary cancer is 7.8/100,000, higher than 3/100,000 in China (4). A significant portion of patients were diagnosed with advanced disease coexisting with chronic diseases, poor performance status, and systemic immunosuppressive states due to cold immune microenvironment characteristics (5, 6). There are limited therapies for advanced pancreatic and biliary cancer; thus, the prognosis is extremely poor. Consequently, these cancer patients may be more vulnerable to COVID-19 than other populations. A recent study reported a higher risk of severe outcomes in patients with hematologic cancer, lung cancer, or metastatic cancer after infection with COVID-19 (7, 8). However, there has been no systematic evaluation of the effects that the Omicron variant has induced in patients with this leading deadly cancer in a representative population till now.

Our team is dedicated to research on the prognosis and outcome of advanced pancreatic and biliary cancer, and we have paid more attention to the disease changes in the above vulnerable population after Omicron infection since the COVID-19 outbreak in Shanghai. Here we started an observational study to describe the clinical characteristics and outcomes of patients affected by the Omicron variant with or without pancreatic and biliary cancer. We evaluated immune response functions based on lymphocyte subtypes and investigated the role of inflammatory cytokines on the course and outcomes in Omicron-variant-infected cancer patients. The findings indicated that patients with pancreatic and biliary cancer appeared more vulnerable to an Omicron outbreak. The information and insights provided in this study will help us better focus on the poor prognosis of vulnerable populations representatively with pancreatic and biliary cancer after Omicron infection, improve our understanding of the dynamic evolution of clinical and immunological indexes in Omicron-infected patients with pancreatic and biliary cancer, and thus offer useful evidence for clinicians for the management of these vulnerable populations from a welfare view for future pandemics.

## 2 Methods

### 2.1 Study design and participants

**Figure 1:**
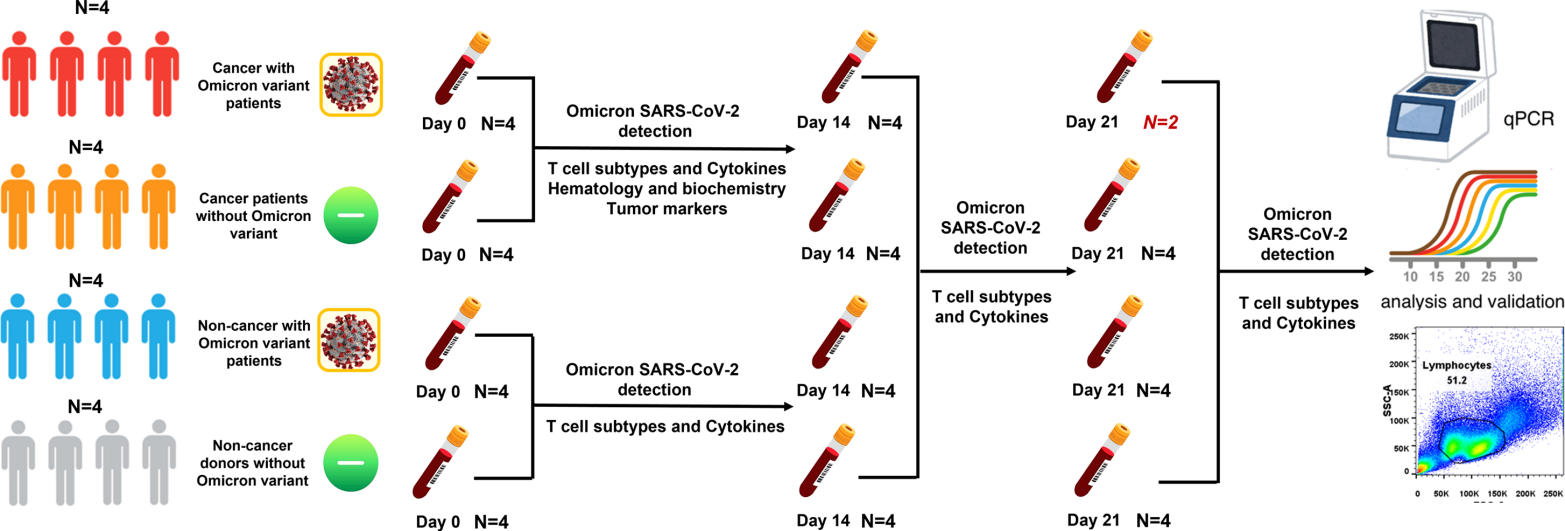
Study profile. There were four groups in this study. Group 1: cancer patients with omicron infection; Group 2: cancer patients without omicron infection; Group 3: non-cancer patients with omicron infection; and Group 4: non-cancer-afflicted subjects without omicron infection.

From 15 April to 31 May 2022, we conducted an observational study in four Omicron-infected patients with advanced pancreatic and biliary cancer (Group 1) in the Oncology Department of the Seventh People’s Hospital of the Shanghai University of Traditional Chinese Medicine (TCM). Omicron infection was clinically defined, referring to WHO diagnostic criteria. At three time points (Days 0, 7, and 14 after the diagnosis of Omicron infection), lymphocyte subsets, cytokines, white blood cell count, blood biochemistry, and tumor markers were detected for Omicron-infected cancer patients; a 45-day clinical observation was conducted, and the data of tumor diagnosis, clinical disease course, and previous tumor treatment were collected. To clarify the effects of Omicron infection on the medical condition of patients with advanced pancreatic and biliary cancer, four non-Omicron-infected cancer patients (Group 2), four Omicron-infected non-cancer-afflicted subjects (Group 3), and four non-Omicron-infected non-cancer-afflicted subjects (Group 4) were included in this study at the same time to serve as controls, and their blood samples were collected at the same time points to detect lymphocyte subsets and cytokines. The subjects in Groups 1 and 2 were paired by age, sex, body mass index (BMI), pathological diagnosis, tumor stage, and tumor co-morbidities. Omicron-infected patients were defined as patients diagnosed with advanced pancreatic and biliary cancer using pathological or clinical evidence whose RT-PCR test result was positive. The non-cancer-afflicted subjects were enrolled by caregivers. All 16 subjects had no history of COVID-19 infection. The vaccination status of 16 subjects is shown in **Table 1**. This study was approved by the Ethics Committee of the Seventh People’s Hospital of Shanghai University of TCM and is under the jurisdiction of Shanghai epidemic prevention policies, regulations, and laws. In the Seventh People’s Hospital of the Shanghai University of TCM, all subjects received face-to-face interviews and signed written informed consent forms (**Figure 1**).

**Table 1:** Vaccination status of 16 subjects.

Groups	Group 1 Omicron-infected cancer patients				Group 2 Non-infected cancer patients				Group 3 Infected healthy subjects				Group 4 Non-infected healthy subjects			
Age (years)	70	73	57	51	63	49	66	60	72	68	46	50	55	41	57	59
Vaccination status (doses)	1	0	3	1	1	2	4	2	0	0	3	0	2	3	0	3

### 2.2 Data collection

We retrospectively analyzed the clinical diagnosis and treatment records, nursing records, laboratory examination results, and imaging examination data of Omicron-infected and control cancer patients. The admission data for these subjects involved the period of 7 February to 31 May 2022. The epidemiological, clinical, laboratory, and imaging examination data of patients were collected and entered into electronic case report forms (CRFs). The CRFs were reviewed and checked by independently two investigators. If incapable of being obtained from electronic medical records, epidemiological and clinical symptom data were determined by the investigators through communication with patients and their families.

### 2.3 Procedures

In this study, pharyngeal swab samples and blood samples of subjects were collected and then sent to the standardized SARS-CoV-2 testing laboratory. The pharyngeal swab samples were tested using fluorescent RT-PCR approved by the National Medical Products Administration (NMPA, former: State Food and Drug Administration (SFDA)). The RT-PCR test results indicated SARS-CoV-2 Omicron Variant (Omicron) in the respiratory samples.

The primary examination included whole blood cell count, blood biochemistry (liver function, renal function, and electrolytes), tumor markers, lymphocyte subsets, cytokines, RT-PCR, and antibody tests of biliary samples for cancer patients, only lymphocyte subsets and cytokines for non-cancer patients, as well as additional RT-PCR and serum antibody tests of pharyngeal swab samples for Omicron-infected patients.

In view of previous studies and explorations on COVID-19 treatment and considering the clinical symptoms of Omicron infection, Omicron-infected subjects were administrated with Lianhua Qingwen capsules 1.4 g tid p.o. Oxygen inhalation support (e.g., nasal catheter oxygen inhalation) was provided upon blood oxygen saturation (SaO_2_). The pharyngeal swab samples of subjects diagnosed as Omicron infection were daily tested by RT-PCR till discharge, death, or a negative RT-PCR result.

### 2.4 Cytokine and lymphocyte subsets measurement

To describe the changes of lymphocyte subsets and cytokines in the body at different stages of disease after Omicron infection, cytokines (IL-1β, IL-2, IL-4, IL-5, IL-6, IL-8, IL-10, IL-12p70, IFN-γ, IFN-α, and TNF-α), lymphocyte subsets (total T-cell%, total T-cell count, helper/inducer T-cell%, helper/inducer T-cell count, cytotoxic T-cell%, cytotoxic T-cell count, helper T-cell/cytotoxic T cell, total B cell%, total B cell count, NK cell%, and NK cell count) and serum antibodies (IgG antibody and IgM antibody) of subjects in four groups were tested on Days 0, 7, and 14 after the diagnosis of Omicron infection. T-cell subsets were detected using BD Bioscience kits, and cytokines were detected using Raisecare kits. The above test results of subjects in the other three groups were used as controls for cross-comparison.

### 2.5 Statistical analysis

The plasma levels of cytokines and lymphocyte subsets in four groups were compared with standard parametric and non-parametric tests, and then the polygram and histogram were plotted to visualize the overall levels and dynamic changes of cytokines and lymphocyte subsets in different groups. The quantitative data were presented as mean ± standard deviation or inter-quartile range (IQR). The qualitative data were expressed as percentages (%). GraphPad Prism 9.3.1 software and SAS 9.4 software were used for analysis. P <0.05 indicated a statistically significant difference.

### 2.6 Role of the funding source

The funders of the study had no role in the study design, data collection, data analysis, data interpretation, or writing of the report.

## 3 Results

### 3.1 Patient characteristics

A total of 16 subjects in the Second Department of Oncology of the Seventh People’s Hospital of Shanghai University of TCM were included from 15 April to 31 May 2022. Since the diagnosis of Omicron infection, 45-day clinical observation has been performed. The observation results showed that three subjects died separately at Days 11, 25 (Day 9 after negative conversion), and 13 after Omicron infection, and they were all Omicron-infected patients with advanced cancer. In the 16 subjects, there were nine (56%) males and seven (44%) females; there were four (100%) males in Omicron-infected cancer patient group and three (75%) males and one (25%) female in non-Omicron-infected cancer patient group, four (100%) females (the caregivers of cancer patients with positive Omicron infection) in Omicron-infected healthy subject group, as well as two (50%) males and two (50%) females in non-Omicron-infected healthy subject group. The age and BMI of subjects in the four groups were 62.75, 59.5, 59, and 53 years, as well as 23.58 kg/m^2^, 21.6 kg/m^2^, 25.83 kg/m^2^, and 23.32 kg/m^2^, respectively. All subjects had no history of COVID-19 infection. The detailed information on subjects in the four groups is shown in **Table 2**.

**Table 2:** Patient’ characteristics and clinical presentations after omicron infected.

Characteristic	Group 1 (N = 4)	Group 2 (N = 4)	Group 3 (N = 4)	Group 4 (N = 4)
Age, years	62.75	59.50	59.00	53.00
Sex				
Female	0	1 (25%)	4 (100%)	2 (50%)
Male	4 (100%)	3 (75%)	0	2 (50%)
BMI, kg/m²	23.58	21.60	25.83	23.32
Tumor type				
Biliary cancer	3 (75%)	4 (100%)	–	–
Pancreatic cancer	1 (25%)	0	–	–
TNM				
I	0	0		
II	1 (25%)	0		
III	2 (50%)	2 (50%)		
IV	1 (25%)	2 (50%)		
Comorbidities				
Jaundice	2 (50%)	4 (100%)	–	–
Hypoproteinemia	4 (100%)	1 (25%)	–	–
Electrolyte disturbance	3 (75%)	3 (75%)	–	–
Hepatic insufficiency	3 (75%)	0	–	–
Tumor metastasis	1 (25%)	3 (75%)	–	–
Anemia	4 (100%)	2 (50%)	–	–
Hemorrhage	1 (25%)	0	–	–
Pleural effusion	1 (25%)	1 (25%)	–	–
Ascites	3 (75%)	2 (50%)	–	–
Thrombosis	1 (25%)	0	–	–
Smoking	2 (50%)	2 (50%)	0	1 (25%)
Drinking	2 (50%)	1 (25%)	0	0
Tumor treatment				
Iodine Implant Surgery	1 (25%)	4 (100%)	–	–
Surgery	2 (50%)	2 (50%)	–	–
Targeted therapy	1 (25%)	3 (75%)	–	–
Immunotherapy	1 (25%)	2 (50%)	–	–
Chemotherapy	3 (75%)	0	–	–
Radiation therapy	0	1 (25%)	–	–
Clinical presentation				
Atypical	3 (75%)	–	2 (50%)	–
Chest pain	0	–	0	–
Cough	1 (25%)	–	0	–
Dyspnea	1 (25%)	–	0	–
Fever	1 (25%)	–	0	–
Other flu-like symptom	1 (25%)	–	2 (50%)	–

"-"on behalf of non-tumor patients or non-Omicron infected patients without relevant examination.

### 3.2 Disease course and outcome

Patient No. 1 was diagnosed with cholangiocarcinoma (stage: cT3N0M0, IIB) over 4 months ago without any underlying diseases. On 26 February 2022, the patient was admitted to the hospital, and the examination indicated common duct obstruction with obstructive jaundice, hypoproteinemia, and electrolyte disturbance. The patient received nasobiliary drainage, artificial liver support, and symptomatic support treatment during hospitalization (**Supplementary Figure 1**). On 15 April 2022, the RT-PCR test results of the pharyngeal swab sample and the bile sample were positive, and the antibody test results of the serum sample and the bile sample were negative for IgM antibodies and negative for IgG antibodies (**Table 3**). After Omicron infection, the patient presented cough and expectoration without other common symptoms or signs of COVID-19 infection and then received antiviral treatment with Lianhua Qingwen capsules. Also, symptomatic support treatment was performed for jaundice, hypoproteinemia, and electrolyte disturbance, and the patient’s general state was stable. On 4 May 2022, the RT-PCR test result of the pharyngeal swab sample was negative, and thereafter, such RT-PCR tests were repeated continuously for 14 days, all with a negative result (**Table 3**).

**Table 3:** RT-PCR and IgG and IgM detection of four groups.

			Day 0						Day 7			Day 14			Day 16	Day 19
			Bile			Venous blood		Pharyngeal swab	Venous blood		Pharyngeal swab	Venous blood		Pharyngeal swab	Pharyngeal swab	Pharyngeal swab
			IgG	IgM	RT-PCR	IgG	IgM	RT-PCR	IgG	IgM	RT-PCR	IgG	IgM	RT-PCR	RT-PCR	RT-PCR
Group 1	Patient 1		−	−	+	−	−	+	−	−	+	+	−	+	+	−
	Patient 2		−	−	+	−	−	+	−	−	+	NA	NA	NA	NA	NA
	Patient 3		−	−	+	±	−	+	+	−	+	+	+	+	−	−
	Patient 4		−	−	+	−	−	+	−	−	+	NA	NA	NA	NA	NA
Group 2	Patient 5		NA	NA	NA	−	−	−	−	−	−	−	−	−	−	−
	Patient 6		NA	NA	NA	−	−	−	−	−	−	−	−	−	−	−
	Patient 7		NA	NA	NA	−	−	−	−	−	−	−	−	−	−	−
	Patient 8		NA	NA	NA	−	−	−	−	−	−	−	−	−	−	−
Group 3	Patient 9		NA	NA	NA	−	−	+	−	−	+	–	–	+	–	–
	Patient 10	NA		NA	NA	−	−	+	−	−	+	NA	NA	+	+	–
	Patient 11		NA	NA	NA	+	−	+	+	−	+	+	–	+	–	–
	Patient 12		NA	NA	NA	−	−	+	−	−	+	NA	NA	+	+	–
Group 4	Patient 13		NA	NA	NA	−	−	−	−	−	−	–	–	–	–	–
	Patient 14		NA	NA	NA	±	−	−	±	−	−	±	–	–	–	–
	Patient 15		NA	NA	NA	−	−	−	−	−	−	–	–	–	–	–
	Patient 16		NA	NA	NA	±	−	−	+	−	−	+	–	–	–	–

"+"on behalf of positive, "±"on behalf of Weakly positive, " -" on behalf of negative, "NA" on behalf of tumor patients with non-Omicron infection were not tested, or died and could not be tested.

Patient No. 2 was diagnosed with hilar cholangiocarcinoma (stage: cT3N0M0, IIIA) over 4 months ago and previously underwent a radical gastrectomy. On 2 March 2022, the patient was admitted to the hospital for biliary drainage and replacement but received symptomatic support treatment during hospitalization due to the lack of a decrease in jaundice-related indexes and the co-morbidities of hepatic cirrhosis, cholecystolithiasis, hypoproteinemia, ascites, splenomegaly, hepatic insufficiency, and hepatic encephalopathy (**Supplementary Figure 2**). On 15 April 2022, the RT-PCR test results of the pharyngeal swab sample and the bile sample were positive, while the antibody test results of the serum sample and the bile sample were negative for IgM antibodies and negative for IgG antibodies (**Table 3**). After Omicron infection, the patient had no symptoms or signs of COVID-19 infection and received antiviral treatment with Lianhua Qingwen capsules but presented aggravation in the medical condition. On 20 April 2022, the ultrasonography indicated 1,000 ml of ascites, and the test results showed severe jaundice and hepatic insufficiency. After symptomatic treatment, the patient had no improvement and then died clinically on 26 April 2022. The cause of death was multiple organ failure, which was related to underlying diseases.

Patient No. 3 was diagnosed with pancreatic cancer (stage: cT3N2M1, IV) over 2 months ago (**Supplementary Figure 3**). On 12 March 2022, the patient was admitted to the hospital. On 24 March 2022, the patient received chemotherapy with the GEMOX regimen (gemcitabine 1.8 g + oxaliplatin 150 mg) after diagnosis. On Day 4 after chemotherapy, the patient felt palpitations and chest distress, and the examination indicated multiple embolisms in the distal right main pulmonary artery and bilateral pulmonary arteries and deep venous thrombosis of the lower limbs. Considering the co-morbidities, including secondary malignancies of the liver, bones, and lymph nodes, hepatic insufficiency, hypoproteinemia, ascites, and hydrothorax, inferior vena cava filter implantation, and symptomatic support treatment, were performed during hospitalization. On 15 April 2022, RT-PCR test results of pharyngeal swab sample and bile sample were positive, the antibody test results of serum sample were weak positive IgM antibody and negative IgG antibody, and those of bile sample were negative IgM antibody and negative IgG antibody (**Table 3**). After Omicron infection, the patient presented cough, expectoration, and throat pain, without other symptoms or signs of COVID-19 infection, and then received antiviral treatment with Lianhua Qingwen capsules. On 30 April 2022, the RT-PCR test result of the pharyngeal swab sample was negative, and thereafter the daily retest results were all negative. The patient had a poor general state. On 7 May 2022, ultrasonography indicated massive ascites and a poor conscious state, with liver failure, renal failure and electrolyte disturbance, and the daily urine volume was 500 ml; after symptomatic treatment, there was no improvement. On 9 May 2022, patient’s blood pressure (78/47 mmHg) and SaO_2_ were both decreased, and SaO_2_ was 89%–93% under high-flow oxygen inhalation. On 10 May 2022, the blood pressure and heart rate were further decreased, and the patient died clinically. The cause of death was multiple organ failure, which was related with underlying diseases.

Patient 4 was diagnosed with cholangiocarcinoma (stage: rT2N1M0, IIIB) over 2 years ago (**Supplementary Figure 4**). On 7 February 2022, the patient was admitted to the hospital, and the examination and test results indicated gastrointestinal hemorrhage, hemobilia, biliary infection, hypoproteinemia, hypokalemia, ascites, and liver abscess. During hospitalization, the patient underwent percutaneous transhepatic cholangial drainage, liver abscess puncture drainage, abdominal puncture drainage, inferior mesenteric artery embolization, and symptomatic support treatment. On 15 April 2022, RT-PCR test results of pharyngeal swab sample and bile sample were positive, the antibody test results of serum sample and bile sample were negative IgM antibody and negative IgG antibody (**Table 3**). After Omicron infection, the patient had a poor general state, intermittent chest distress, shortness of breath, decreased SaO_2_ (min. 85%), fever, cough, expectoration, and restlessness (which all could be transiently relieved after symptomatic support treatment), without other symptoms and signs of COVID-19 infection, and then received antiviral treatment with Lianhua Qingwen capsules. On 28 April 2022, the patient experienced sudden chest distress, shortness of breath, and decreased SaO_2_ (55%–59%), and these symptoms were not improved by symptomatic treatment; thus, the patient died clinically. The cause of death was respiratory failure, which was related with Omicron infection.

### 3.3 Co-morbidities

Before the diagnosis of Omicron infection, there were two cases of jaundice, four cases of hypoproteinemia, three cases of electrolyte disturbance, three cases of hepatic insufficiency, one case of metastasis, four cases of anemia, one case of upper gastrointestinal hemorrhage, one case of hydrothorax, three cases of ascites, and one case of thrombus in Omicron-infected cancer patients. All non-Omicron-infected cancer patients had advanced biliary cancer, in which there were four cases of jaundice, one case of hypoproteinemia, three cases of electrolyte disturbance, three cases of metastasis, two cases of anemia, one case of hydrothorax, and two cases of ascites. The patients’ previous tumor treatments are shown in **Table 2** (before the diagnosis of Omicron virus infection). After the diagnosis of Omicron infection, all Omicron-infected cancer patients and three non-Omicron-infected cancer patients did not receive anti-tumor treatment, and only one non-Omicron-infected cancer patient received targeted therapy (**Table 2**).

### 3.4 Detection of lymphocyte subsets

**Figure 2:**
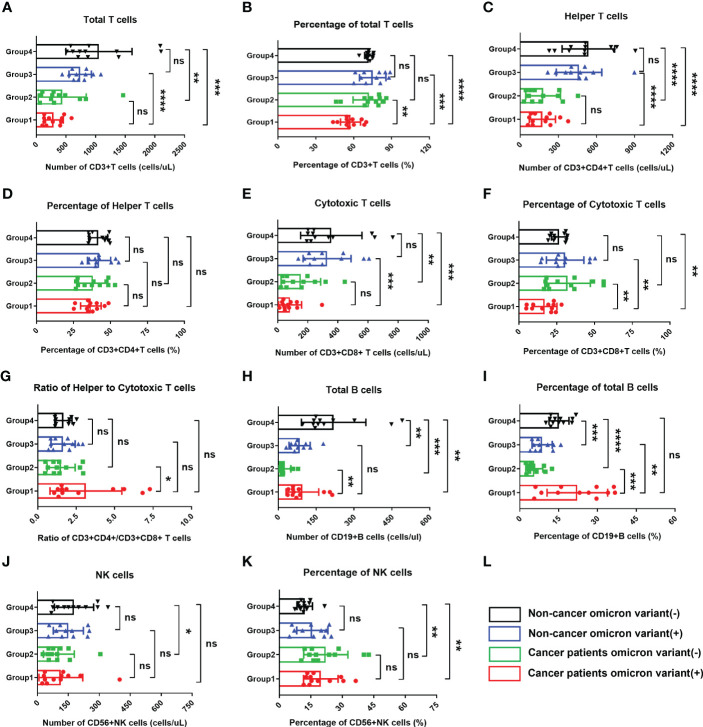
Comparisons of lymphocyte subsets in four groups of participants. The comparisons of count of total T cells in four groups during clinical observation **(A)**, percentage of total T cells **(B)**, count of helper T lymphocytes **(C)**, percentage of helper T lymphocytes **(D)**, count of cytotoxic T cells **(E)**, percentage of cytotoxic T cells **(F)**, ratio of helper to cytotoxic T lymphocytes **(G)**, count of total B cells **(H)**, percentage of total B cells **(I)**, count of NK cells **(J)**, percentage of NK cells **(K)**. **(L)** Black, non-cancer omicron variant (−); Blue, non-cancer omicron variant (+); Green, cancer patients omicron variant (−); Red, cancer patients omicron variant (+). Points in each bar represent 4 subjects’ data from different time points. ns, not statistically significant. *p <0.05, **p <0.01, ***p <0.001, ****p <0.0001.

Based on previous study reports, lymphopenia (an abnormal decrease in the number of lymphocytes) is an important characteristic of COVID-19 infection (9). To fully evaluate the body immunity of Omicron-infected patients with advanced pancreatic and biliary cancer, we compared the overall test results of lymphocyte subsets among four groups in this study (**Figure 2**). The findings showed that total T cell% (**Figure 2B**) and CD8^+^ T cell% (**Figure 2F**) in Omicron-infected cancer patients were lower than those in non-Omicron-infected cancer patients (both P <0.01), while CD4^+^T/CD8^+^T (**Figure 2G**), total B cell count (**Figure 2H**), and total B cell% (**Figure 2I**) were higher (P <0.05, P <0.01, and P <0.001). Compared with Omicron-infected non-cancer-afflicted subjects, total T-cell count (**Figure 2A**), total T cell% (**Figure 2B**), CD4^+^ T-cell count (**Figure 2C**), and CD8^+^ T-cell count (**Figure 2E**) in Omicron-infected cancer patients were decreased (P <0.0001, P <0.001, P <0.0001, and P <0.001), while total B cell% (**Figure 2I**) was increased (P <0.01).

**Figure 3:**
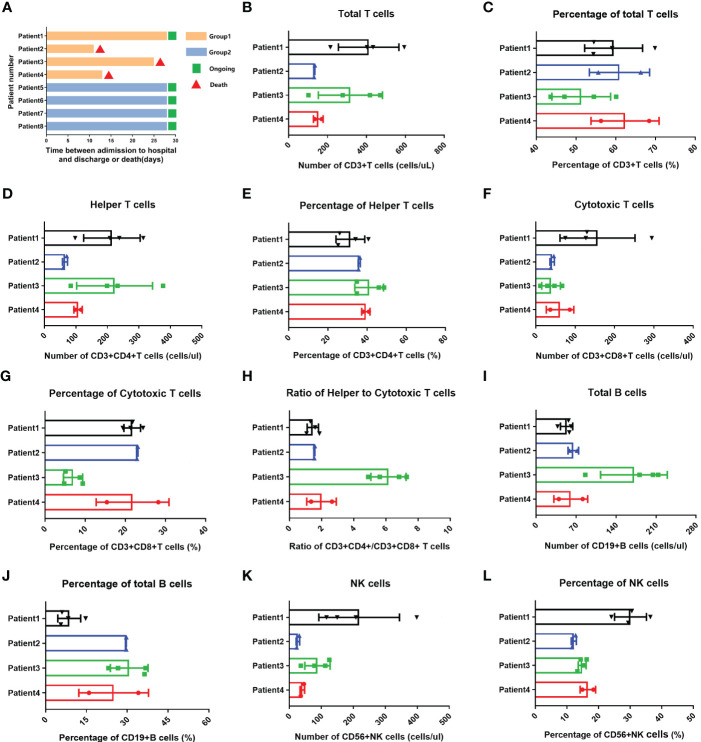
Swimmer plots for patient survival and comparisons of lymphocyte subsets in four cancer patients with omicron infection. **(A)** Of cancer patients with omicron infection, Patient 1 survived, but Patients 2, 3, and 4 died after Omicron virus infection on the 11th, 25th (9 days after the virus test was negative), and 13th. Of the cancer patients without Omicron infection, Patients 5, 6, 7, and 8 all survived. The comparisons of count of total T cells in four cancer patients with Omicron virus infection during clinical observation **(B)**, percentage of total T cells **(C)**, count of helper T lymphocytes **(D)**, percentage of helper T lymphocytes **(E)**, count of cytotoxic T cells **(F)**, percentage of cytotoxic T cells **(G)**, ratio of helper to cytotoxic T lymphocytes **(H)**, count of total B cells **(I)**, percentage of total B cells **(J)**, count of NK cells **(K)**, percentage of NK cells **(L)**.

Of four Omicron-infected cancer patients, Patient No. 1 still survived, while Patients Nos. 2, 3, and 4 died on Days 11, 25 (Day 9 after negative conversion), and day 13 after Omicron infection, respectively (**Figure 3A**). To further evaluate the relationship between body immunity and disease course and prognosis in Omicron-infected patients with advanced pancreatic and biliary cancer, we compared the test results of lymphocyte subsets among four Omicron-infected cancer patients, only one of whom survived (**Figure 3**). The findings revealed that CD4^+^T/CD8^+^T (**Figure 3H**), B cell count (**Figure 3I**), and total B cell% (**Figure 3J**) of Patient No. 1 were lower than those of Patient No. 3, while CD8^+^T cell% (**Figure 3G**) and NK cell% (**Figure 3L**) were greater. Compared with Patient No. 2, the total B cell% (**Figure 3J**) of Patient No. 1 was declined, while NK cell% (**Figure 3L**) was elevated. NK cell% (**Figure 3L**) of Patient No. 1 was greater than that of Patient No. 4 (P <0.05). These findings indicated a poor prognosis in Omicron-infected patients with advanced pancreatic and biliary cancer who had decreased T-cell subsets and immunodeficiency.

### 3.5 Detection of cytokines

**Figure 4:**
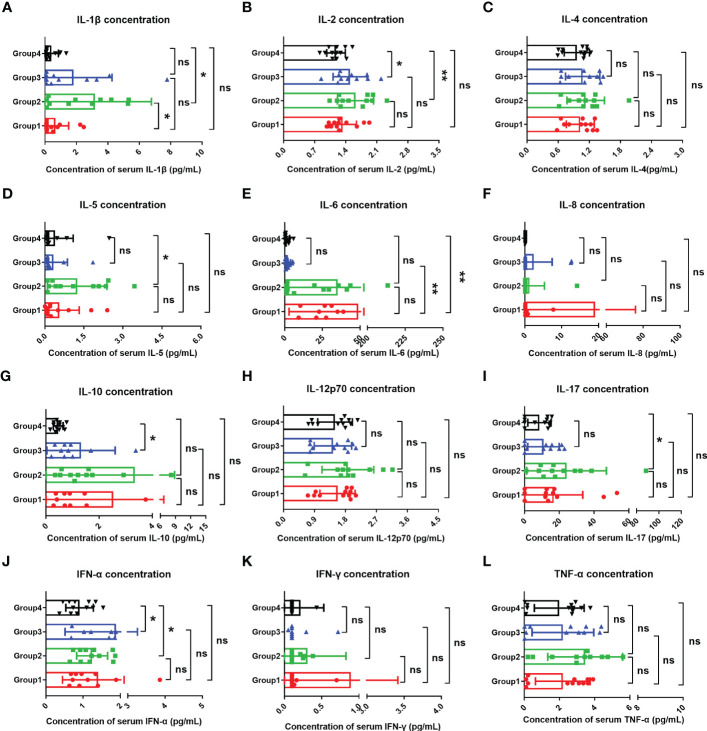
Comparisons of cytokines in four groups. The comparisons of overall IL-1β **(A)**, IL-2 **(B)**, IL-4 **(C)**, IL-5 **(D)**, IL-6 **(E)**, IL-8 **(F)**, IL-10 **(G)**, IL-12p70 **(H)**, IL-17 **(I)**, IFN-α **(J)**, IFN-γ **(K)**, and TNF-α **(L)** between four groups during clinical observation. Points in each bar represent four subjects’ data from different time points. ns, not statistically significant. *p <0.05, **p <0.01.

**Figure 5:**
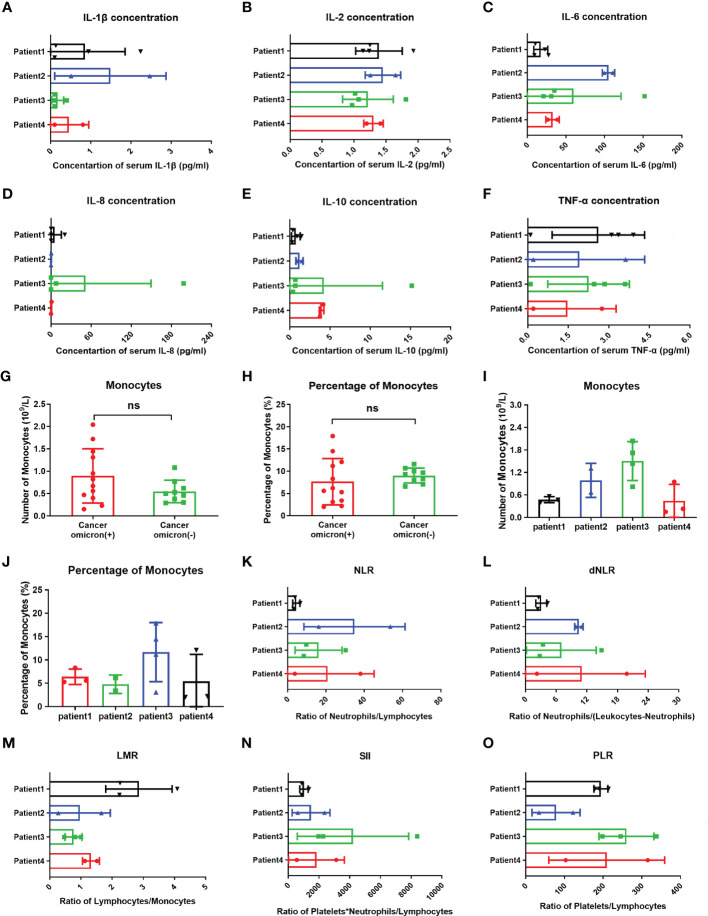
Comparisons of cytokines, monocytes, and inflammation indexes in four cancer infected patients and monocytes between cancer patients with and without omicron infection. The comparison of overall IL-1β **(A)**, IL-2 **(B)**, IL-6 **(C)**, IL-8 **(D)**, IL-10 **(E)**, TNF-α **(F)**, count of monocytes **(I)**, percentage of monocytes **(J)**, NLR **(K)**, dNLR **(L)**, LMR **(M)**, SII **(N)**, and PLR **(O)** between four cancer patients with Omicron virus infection during clinical observation. **(G, H)** The comparison of monocyte counts and monocyte percentages between cancer patients with and without Omicron infection during clinical observation. Points in each bar represent four subjects’ data from different time points. ns, not statistically significant.

Studies have suggested that when infecting the epithelial cells, COVID-19 SARS-CoV-2 will be recognized by macrophages and adjacent endothelial and epithelial cells, causing the secretion of massive proinflammatory cytokines (10). To evaluate the effects of Omicron infection on the secretion of proinflammatory cytokines in patients with advanced pancreatic and biliary cancer, we detected the serum cytokine levels of subjects in four groups and then performed a comparative analysis (**Figure 4**). The results demonstrated that IL-1β (**Figure 4A**) and IL-6 (**Figure 4E**) in Omicron-infected cancer patients were lower than those in non-Omicron-infected cancer patients (P <0.05) and higher than those in Omicron-infected non-cancer-afflicted subjects (P <0.01), respectively. As shown by the clinical observation, four Omicron-infected cancer subjects had different outcomes: Patient No. 1 still survived, while Patients Nos. 2, 3, and 4 died on Days 11, 25 (Day 9 after negative conversion), and 13 after Omicron infection, respectively. To evaluate the relationship between the secretion level of proinflammatory cytokines and the disease outcome in Omicron-infected patients with advanced pancreatic and biliary cancer, we compared the serum cytokine test results of Patient No. 1 and the remaining three patients (**Figure 5**). The findings indicated that the secretion levels of IL-6 (**Figure 5C**) and IL-10 (**Figure 5E**) in Patient No. 1 were lower than those in Patients Nos. 2 and 4, respectively. These findings indicated T-cell exhaustion and immunodeficiency in Omicron-infected patients with advanced pancreatic and biliary cancer.

### 3.6 Monocyte detection

Studies have shown that after invading human body, SARS-CoV-2 will infect monocytes and macrophages, and these two types of cells die of cellular inflammatory necrosis (i.e., pyroptosis) and thus release strong inflammatory warning signals. In COVID-19 patients, about 6% of monocytes die of pyroptosis (11). To fully evaluate the immune state of Omicron-infected patients with advanced pancreatic and biliary cancer, we detected and compared peripheral blood monocytes in four Omicron-infected and four non-Omicron-infected patients with advanced pancreatic and biliary cancer (**Figure 5**). As shown by the results, there was no significant difference in monocyte count (**Figure 5G**) and monocyte% (**Figure 5H**) between the two groups (P >0.05). In four Omicron-infected cancer patients, however, the monocyte count (**Figure 5I**) of surviving Patient No. 1 was smaller than that of Patient No. 3.

### 3.7 Inflammatory indexes

Previous studies have revealed that the neutrophil to lymphocyte ratio (NLR), derived neutrophil-to-lymphocyte ratio (dNLR), lymphocyte to monocyte ratio (LMR), platelet to lymphocyte ratio (PLR), and systemic immune-inflammatory index (SII) is closely related to the development and prognosis of tumors. Numerous studies completed after the COVID-19 outbreak suggested that these inflammatory indexes also have a significant correlation with the prognosis of COVID-19 patients (12). In this study, the comparison of inflammatory indexes between Omicron-infected and non-Omicron-infected cancer patients showed no significant differences (**Table 4**). To further explore the correlation between inflammatory indexes and prognosis, we compared the inflammatory indexes among four Omicron-infected cancer patients, only one of whom survived (**Figure 5**). The results showed that the dNLR (**Figure 5L**), PLR (**Figure 5O**), and LMR (**Figure 5M**) of Patient No. 1 were lower than those of Patient No. 2, higher than those of Patient No. 2, and greater than those of Patient No. 3, respectively, and there were no significant differences in the other inflammatory indexes (**Figures 5K–N**).

**Table 4:** Study timepoint analysis of clinical data between cancer patients with Omicron infection and cancer patients without Omicron infection across the three study timepoints.

	Day 0			Day 7			Day 14		
	Group 1	Group 2	P-value	Group 1	Group 2	P-value	Group 1	Group 2	P-value
WBC count × 10^9^/L	11.79	7.09	0.29	13.28	6.72	0.10	21.42	3.81	0.02*
Monocyte count × 10^9^/L	1.19	0.63	0.17	0.73	0.53	0.57	0.85	0.72	0.68
IL-6 pg/ml	47.77	70.53	0.68	42.40	20.12	0.35	22.09	14.62	0.63
RBC count × 10^12^/L	3.18	3.43	0.71	3.23	3.19	0.97	4.23	2.95	0.26
Hb g/L	103.00	109.50	0.76	104.30	103.70	0.98	128.50	98.33	0.38
PLT count × 10^9^/L	163.30	134.00	0.57	197.30	121.00	0.52	316.50	83.00	0.0079*
LYM%	11.58	13.85	0.74	10.05	12.43	0.73	7.65	16.60	0.32
GRAN%	76.50	67.88	0.39	83.45	76.87	0.45	87.45	66.20	0.04*
NLR	17.74	12.40	0.72	16.45	13.30	0.80	18.47	5.77	0.27
dNLR	4.64	3.30	0.63	8.86	3.98	0.36	9.59	2.20	0.16
LMR	1.13	1.41	0.69	1.92	1.39	0.60	1.61	1.15	0.54
PLR	171.30	262.30	0.45	184.50	301.90	0.53	258.30	174.30	0.43
SII 10^9^/L	1,536.00	2,086.00	0.74	1,763.00	2,396.00	0.74	4,630.00	565.60	0.25
Tbil μmol/L	174.80	57.78	0.38	171.20	64.37	0.53	62.15	93.45	0.59
ALT U/L	30.33	21.38	0.11	34.60	32.90	0.80	117.30	54.50	0.08
AST U/L	164.80	39.43	0.34	166.60	35.75	0.43	152.80	64.95	0.14
Urea mmol/L	4.94	4.84	0.95	6.14	3.46	0.39	6.04	2.47	0.28
Cr μmol/L	47.30	49.60	0.79	46.63	33.45	0.22	31.60	27.90	0.67

"*"on behalf of p <0.05. Bold values means on behalf of statistically significant results.

### 3.8 Time-course detection of lymphocyte subsets and cytokines during infection

**Figure 6:**
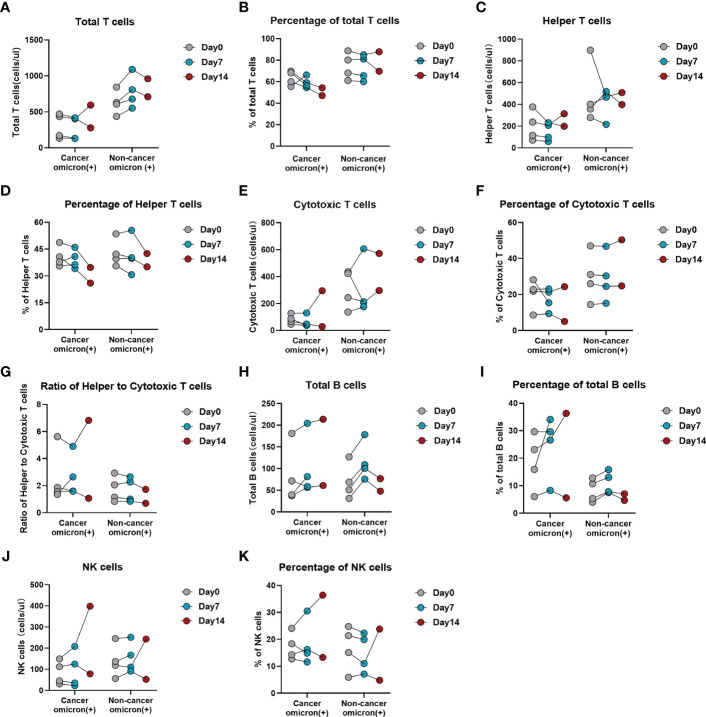
Time-course variations of lymphocyte subsets in Omicron infected patients with or without cancer disease. Group 1 were cancer patients with Omicron infection, and Group 3 were non-cancer patients with Omicron infection. The comparisons of results from three timepoint detections of count of total T cells **(A)**, percentage of total T cells **(B)**, count of helper T lymphocytes **(C)**, percentage of helper T lymphocytes **(D)**, count of cytotoxic T cells **(E)**, percentage of cytotoxic T cells **(F)**, ratio of helper to cytotoxic T lymphocytes **(G)**, count of total B cells **(H)**, percentage of total B cells **(I)**, count of NK cells **(J)**, and percentage of NK cells **(K)** between Groups 1 and 2 during clinical observation.

**Figure 7:**
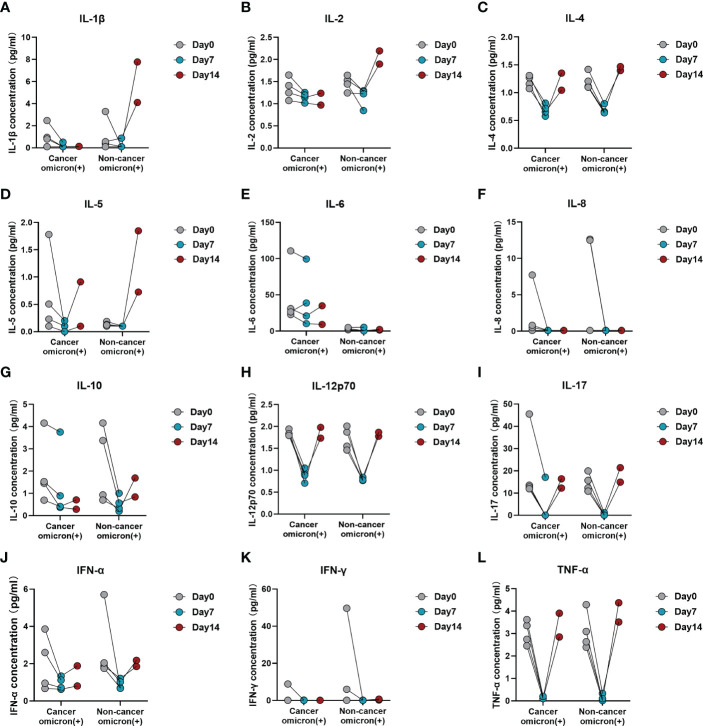
Time-course variations of cytokines in Omicron-infected patients with or without cancer disease. Group 1 were cancer patients with Omicron infection, and Group 3 were non-cancer patients with Omicron infection. The comparison of results from three timepoint detections of IL-1β **(A)**, IL-2 **(B)**, IL-4 **(C)**, IL-5 **(D)**, IL-6 **(E)**, IL-8 **(F)**, IL-10 **(G)**, IL-12p70 **(H)**, IL-17 **(I)**, IFN-α **(J)**, IFN-γ **(K)**, and TNF-α **(L)** between Groups 1 and 2 during clinical observation.

To evaluate the time-course changes in body immunity, we compared the test results of lymphocyte subsets and cytokines over time between Omicron-infected cancer patients and Omicron-infected non-cancer-afflicted subjects. Total T cell% (**Figure 6B**) trended to decrease in Omicron-infected cancer patients but was stable in Omicron-infected non-cancer-afflicted subjects. After Omicron infection, CD4^+^T cell% (**Figure 6D**) had no obvious change in both cancer patients and non-cancer-afflicted subjects at the early stage but trended to decline at the late stage. The B-cell count (**Figure 6H**) of Omicron-infected non-cancer-afflicted subjects was evidently increased at the early stage but trended to decrease at the late stage, while that of Omicron-infected cancer patients trended to increase generally; in the time-course changes of other lymphocyte subsets, there was no significant difference between the two groups (**Figures 6A, C, E–G, I–K**). At the early stage of Omicron infection, IL-1β (**Figure 7A**) trended to decrease in both cancer patients and non-cancer-afflicted subjects; at the late stage, it remained at a low level without apparent fluctuation in cancer patients but trended to a marked increase in non-cancer-afflicted subjects. IL-2 (**Figure 7B**) had a change trend like IL-1β. In both cancer patients and non-cancer-afflicted subjects, IL-4 (**Figure 7C**), IL-5 (**Figure 7D**), IL-12p70 (**Figure 7H**), IL-17 (**Figure 7I**), IFN-α (**Figure 7J**), and TNF-α (**Figure 7L**) all trended to significantly decrease at the early stage and markedly increase at the late stage of Omicron infection. The overall level of IL-6 (**Figure 7E**) in Omicron-infected cancer patients was higher than that in Omicron-infected non-cancer-afflicted subjects, with a decreasing trend at the early stage; however, IL-6 in non-cancer-afflicted subjects was kept at a low level and had no obvious changes in the whole process of Omicron infection. IL-10 (**Figure 7G**) trended to decrease in Omicron-infected cancer patients and Omicron-infected non-cancer-afflicted subjects at the early stage, and its increase in non-cancer-afflicted subjects was higher than that in cancer patients at the late stage. IL-8 (**Figure 7F**) and IFN-γ (**Figure 7K**) had a declining trend in both cancer patients and non-cancer-afflicted subjects at the early stage but no significant changes at the late stage. The above-mentioned serum cytokine time-course changes further indicated that Omicron-infected patients with advanced pancreatic and biliary cancer had T-cell exhaustion and immunodeficiency.

### 3.9 Follow-up assessment of one survivor

Of the four Omicron-infected cancer patients, Patient No. 1 still survived. This patient was admitted to the hospital on 26 February 2022, with the co-morbidities of hypoproteinemia, electrolyte disturbance and severe jaundice (total bilirubin TBIL 618.9 μmol/L). After nasobiliary drainage, artificial liver support, and symptomatic support treatment, there was a significant improvement in liver function. On 4 April 2022, TBIL was tested at 102.3 μmol/L. On 15 April 2022, RT-PCR test results of pharyngeal swab sample and bile sample were positive, and the antibody test results of serum sample and bile sample were negative IgM antibody and negative IgG antibody (**Table 3**). After Omicron infection, the patient presented cough and expectoration without other common symptoms and signs of COVID-19 infection, and then received antiviral treatment with Lianhua Qingwen capsules. During Omicron infection, the patient had normal SaO_2_, a poor mental state, generalized weakness, as well as jaundice, hypoproteinemia, and electrolyte disturbance; after symptomatic treatment, the patient’s general state became stable, and there was no apparent aggravation of symptoms. Time-course detection of lymphocyte subsets and cytokines is shown in **Supplementary Figures 5**, **6**. On 30 April 2022, TBIL was tested at 29.8 μmol/L. On 4 May 2022, RT-PCR test result of pharyngeal swab sample was negative (**Table 3**). On 15 May 2022, the patient’s test results indicated aggravated liver dysfunction with TBIL 81.4 μmol/L; after active symptomatic treatment, the patient had an improved state. On 21 May 2022, TBIL was tested at 37.8 µmol/L. Now this patient has stable disease without jaundice.

## 4 Discussion

The fast spread of COVID-19 has posed a global health threat, especially for cancer patients. A cohort study (13) showed that compared with the COVID-19 Cohort, COVID-19 patients with hematologic, brain, nasopharyngeal, digestive system, and lung malignancies have a significantly higher risk of mortality (44% *vs* 9%, P < .001), while patients with breast and endocrine, genitourinary, and female genital tumors showed a moderate mortality risk that is similar to the COVID-19 Cohort (10% *vs* 9%, P = .85). Several studies have found that hematological cancer and lung cancer were significantly associated with higher mortality (14–16). Infected patients with a history of genitourinary cancer did not have a higher risk of death compared to those without cancer (17). To date, few studies have investigated Omicron-variant infection in cancer patients, especially in tumors derived from the pancreatic and biliary systems, which is the leading cause of death worldwide. In this present study, the choice of patients in Group 1 was very limited, and we tried to make a careful selection of patients to match factors such as age, gender, BMI, etc. We have revealed that patients with advanced pancreatic and biliary cancer infected with the Omicron variant tend to have more severe outcomes compared with asymptomatic carriers without cancer disease or cancer patients with the same pathologic diagnosis and disease status but negative for Omicron infection. Importantly, we aimed to evaluate immune response functions based on lymphocyte subtypes and understand the role of inflammatory cytokines on the course and outcomes in Omicron-variant infected patients with pancreatic and biliary cancer disease.

In late February 2022, a wave of Omicron variant infections rapidly appeared in Shanghai, the biggest city in China. According to the Shanghai Municipal Health Commission, 63,007 Omicron infection cases have been reported with 595 death cases until 31 May 2022. All these new viral genomes in Shanghai were clustered into the SARS-CoV-2 BA.2.2 sub-lineage. Pancreatic and biliary cancers represent the most challenging malignancies facing the oncologist because most patients have been diagnosed with locally advanced, metastatic, or recurrent diseases with poor long-term prognosis (2, 18). Up until now, limited information is known about the outcome of patients with such leading deadliest cancer who contract this Omicron variant, the most highly communicable disease. As well-known, most people with a good physical status only present mild discomfort and even no symptoms after Omicron infection (19). However, the vulnerable population with representatively advanced pancreatic and biliary cancer is just the biggest challenge of Omicron infection management. Importantly, vaccination coverage has remained relatively low for older adults in Shanghai. As reported, the basic full-immunization rate and booster immunization rate of people at the age of ≥60 years were 62% and 38% in Shanghai by 15 April 2022 (1). The overall vaccination coverage has remained low in Chinese people older than 60 years old, especially in elder age patients with a diagnosis of cancer. Although Omicron variant evolves towards less virulent, a higher rate of severe outcomes and mortality cases have been reported in unvaccinated people, especially older adults with chronic diseases (19). Therefore, we conducted this study on pancreatic and biliary cancer patients with coexisting Omicron variant to evaluate the potential effect of Omicron infection on this challenging cancer disease based the data on the period of Shanghai COVID-19 pandemic.

Based on our analysis, Omicron infection patients with pancreatic and biliary cancer tend to have more severe outcomes compared with the noncancer asymptomatic carriers. Three (75%) cancer patients coexisting with Omicron virus died after 11 days, 25 days (9 days after the virus test was negative), and 13 days after diagnosis with a positive infection, respectively. Although COVID-19 is reported to have a relatively low death rate in the general population, patients with pancreatic and biliary cancer and Omicron infection not only have a sharply higher death rate than infected populations without a diagnosis of cancer but also tend to have much higher complications associated with their illness based on our single medical oncology center. These findings suggest that patients with pancreatic and biliary cancer were a much more vulnerable population during the COVID-19 pandemic in Shanghai. Additionally, our study reveals that our alarm is sounded by the high death rate of Omicron-infected patients with advanced pancreatic and biliary cancer as the vulnerable population, and better preventing such a population from Omicron infection is critical to carry out aggressive vaccination and drug treatment. A previous report from Fudan University also indirectly proved poor prognosis of Omicron infection in the vulnerable population. In this report, it was predicted with a model that, in event of no actions during the 6-month simulation period on the scale of China, the Omicron epidemic would result in 5,080,000 hospitalizations and 1,550,000 deaths, and 74.7% of deaths would be “contributed” by the non-vaccinated vulnerable population of ≥60 years (20). Herein, we wish to emphasize that these vulnerable patients with advanced pancreatic and biliary cancer disease should be given positive supportive care by healthcare authorities, service providers, and caregivers during the pandemics. Meanwhile, vaccinated patients should be encouraged to attend standard treatment and receive routine follow-up.

It has been demonstrated an aberrant expression of ACE2 in lung carcinoma compared to normal tissues, regardless of the stage of disease, making lung cancer patients even more vulnerable to COVID-19 (21). Abdul-Jawad et al. (22) proved that patients with hematological cancer and COVID-19 have much less immune activation, high levels of CD8+ T-cell exhaustion, and severe B-cell cytopenia. One of the highlights of our studies is that we focus on the immune functional status during the procedure of infection in pancreatic and biliary cancer-infected patients. Many studies have explored the correlations between SARS-CoV-2 infection severity and lymphopenia since the current global outbreak of COVID-19 (23, 24). One prominent feature of this kind of infection is lymphopenia in elderly patients but not in infected children where the mortality rate is close to zero (25). Notably, lymphocyte counts are prognostic factors in patients with advanced pancreatic or biliary cancer (26). Thus, a better understanding of the potential mechanisms that induce lymphopenia may help to better understand Omicron variant pathogenesis and provide insight into the rational development of therapeutics for such patients with late cancer disease. In this present study, 16 participants underwent lymphocyte subset detection to evaluate T-cell responses. Here, we observed that pancreatic and biliary cancer patients with Omicron infection (average age 63 years) have exhibited significant decreases in T-cell subsets, including total T, helper T, and cytotoxic T cells, with a strikingly increased number of B cells compared with asymptomatic carriers, which dramatically predicted possibly exhausted T cells in infected patients with advanced cancer disease. Moreover, we further observed that lymphopenia was associated with a lower level of IL-1β and a higher level of IL-6 in diseases with poor outcomes, potentially *via* a direct effect of cytokines on T-cell exhaustion. This evidence pointed to possible disruption of T-cell functions, which could not be fully activated in advanced pancreatic and biliary cancer patients after suffering from Omicron infection.

**Figure 8:**
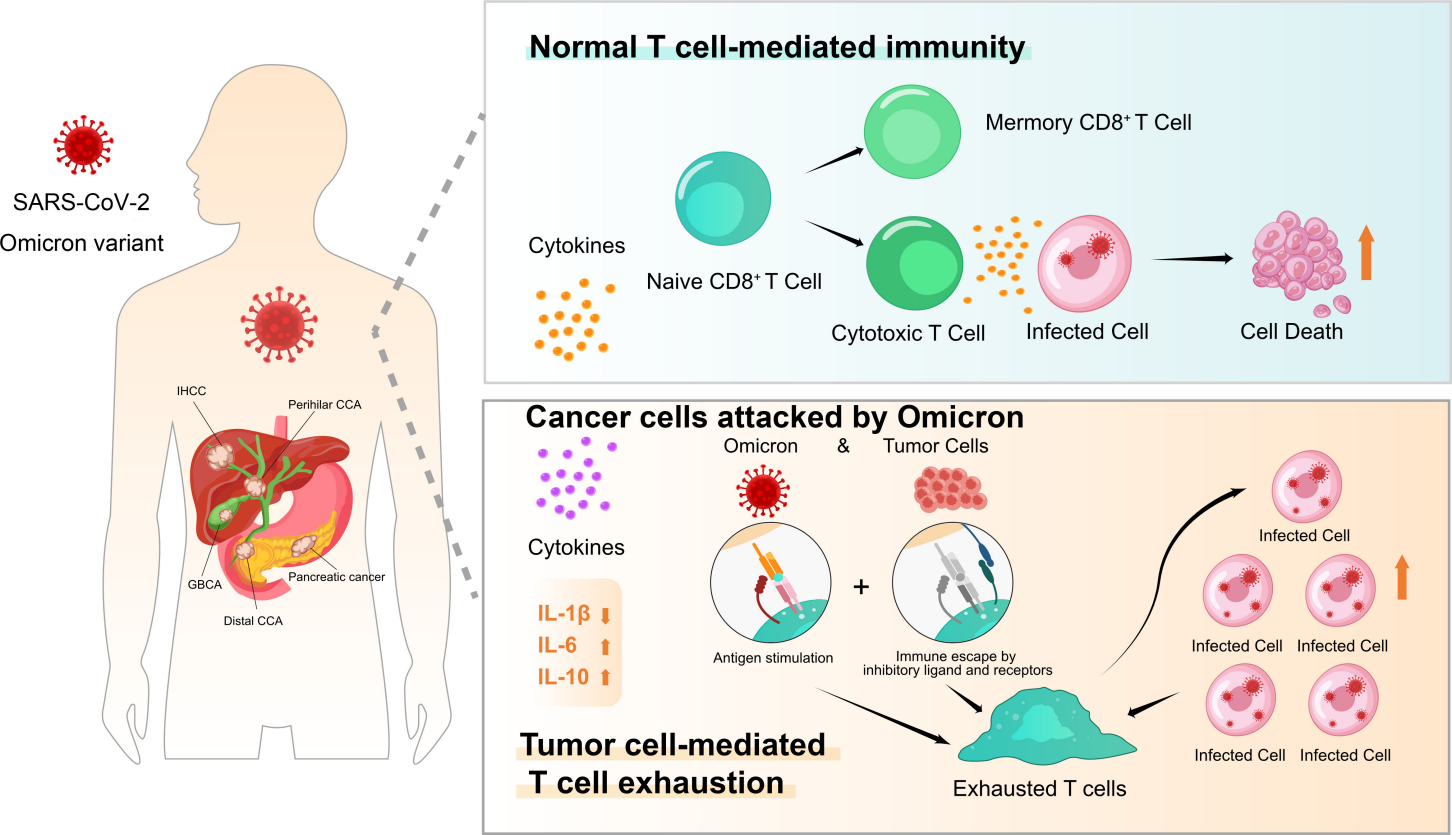
Patients with pancreatic and biliary cancer appeared vulnerable and have severe outcomes to Omicron outbreak in presentation with T-cell exhaustion and immunodeficiency. The effects of normal T-cell-mediated immunity and tumor cell-mediated T-cell exhaustion after Omicron attacks tumor cells on tumor patients are presented.

In addition to T-cell response impairment, uncontrolled inflammation contributes to disease severity in COVID-19 infection (27, 28). The link between chronic inflammation and an increased risk of developing cancer is well established (29). However, little is known about the inflammation-associated immunology of pancreatic and biliary cancer in individuals with Omicron variant infection. Therefore, we performed dynamic monitoring in cancer patients with or without infection. Consistent with this hypothesis, the T-cell immune response was obviously suppressed, while B-cell counts increased gradually in pancreatic and biliary cancer patients who died after Omicron infection within 3 weeks. Moreover, the serum IL-1β level was extremely low during the entire course of virus infection in patients with severe outcomes, whereas IL-6 appeared to maintain higher levels at baseline and gradually increase after infection. Meanwhile, time-course increases in inflammatory scores (dNLR, PLR, and LMR) were found to be correlated with disease severity and a poor prognosis in this study. These results demonstrated that Omicron variant infection could not activate the immune response but further induce T-cell exhaustion due to the cold immune microenvironment in most patients with advanced pancreatic and biliary cancer disease. On the contrary, B-cell counts and percentages of B cell were obviously elevated among infected patients with poor prognoses, which were possible underlying causes for the observed T-cell exhaustion but not the key to recovery based on the available literature. While the role of B cells and anti-Omicron variant antibodies in the recovery process remains to be fully investigated. At present, more concerns are paid to the role of B-cell receptor (BCR) in the preparation of Omicron-specific broad-spectrum antibodies (30, 31), but the features of B cells and their relation with BCRs in patients with pancreatic and biliary cancer are to be discussed (**Figure 8**).

## 5 Conclusion

Overall, this study is the first observational study on Omicron-infected patients with pre-existing advanced pancreatic and biliary cancer, which indicates that lymphopenia and variation levels of certain cytokines, such as serum IL-6 and IL-1β, have been closely associated with severity and poor outcomes in these patients. A complete evaluation of T-cell response and immune status in Omicron-infected patients with basic disease history, co-morbidities, and other various diseases that may influence T-cell response will provide potential opportunities for prevention and therapeutic strategies. In the long term, improving the development of novel, highly efficacious vaccines with long-term immune persistence will be a key priority for older and more vulnerable cancer patients. Patients with pancreatic and biliary cancers with cold tumor characteristics are the vulnerable population that should be highly concerned about clinical practice during the pandemic in the foreseeable future.

## Data availability statement

The original contributions presented in the study are included in the article/**Supplementary Material**. Further inquiries can be directed to the corresponding authors.

## Ethics statement

Written informed consent was obtained from the individual(s) for the publication of any potentially identifiable images or data included in this article.

## Author contributions

TC and JWa contributed to study design, direction, and guidance. TH, LC, JG and SW complete the manuscript, sample detection, clinical data collection, identification and inclusion of patients, and data recording. MM, SW, JY, and HW accomplished the data recording and literature review. JWu performed the statistical analysis. YZ and YC accomplished clinical sample collection and follow-up. All authors listed have made a substantial, direct, and intellectual contribution to the work and approved it for publication.
