# Supplementary figures and images for: Patients with advanced pancreatic and biliary cancer appear vulnerable to SARS-CoV-2 Omicron variant: An observational study during the COVID-19 outbreak in Shanghai
Source: Front Oncol. 2023 Mar 22;13:1115293. doi: 10.3389/fonc.2023.1115293 (PMC10073743; doi:10.3389/fonc.2023.1115293)

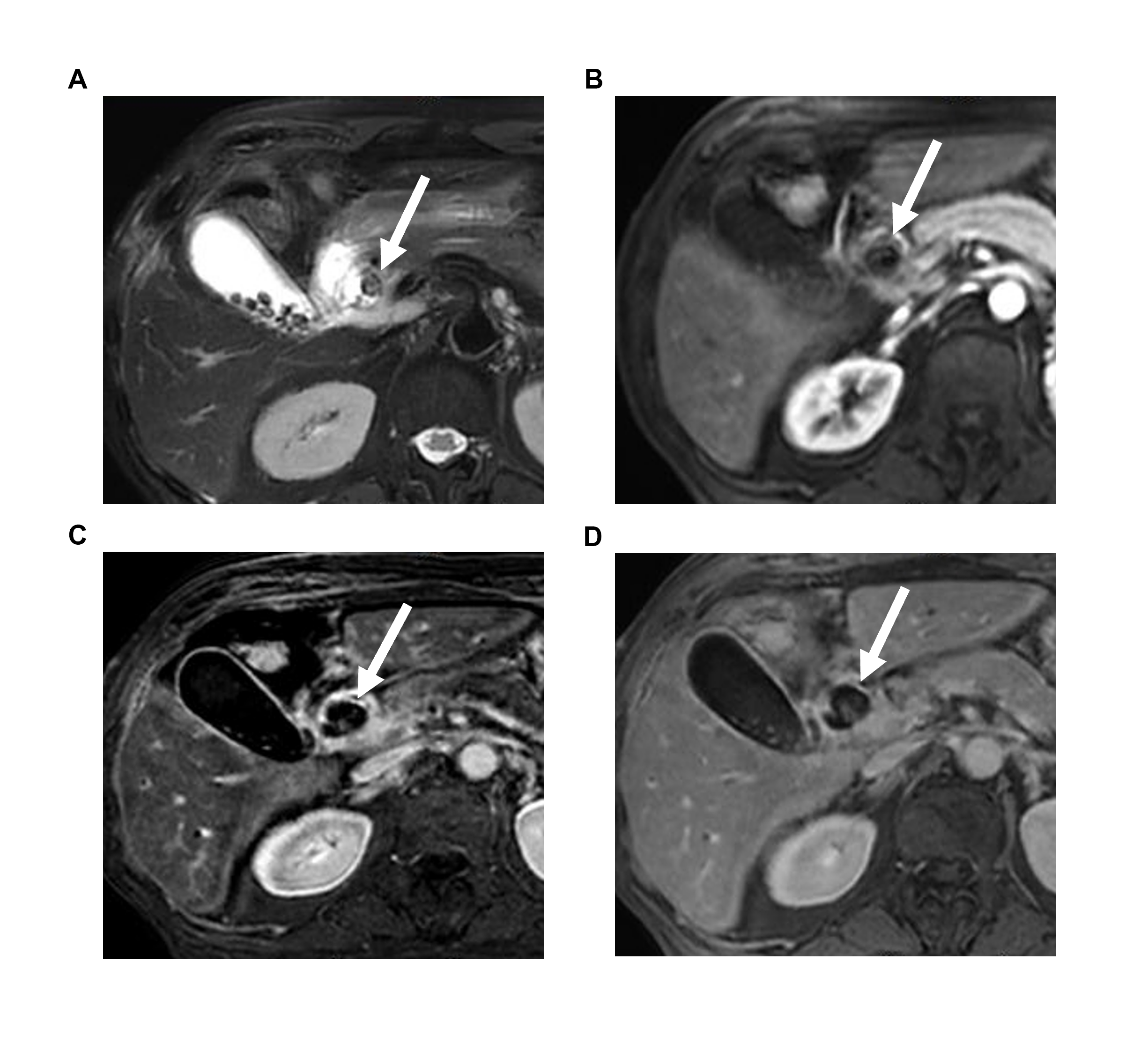

Supplement: Supplementary file 1 [file Image_1.tif]

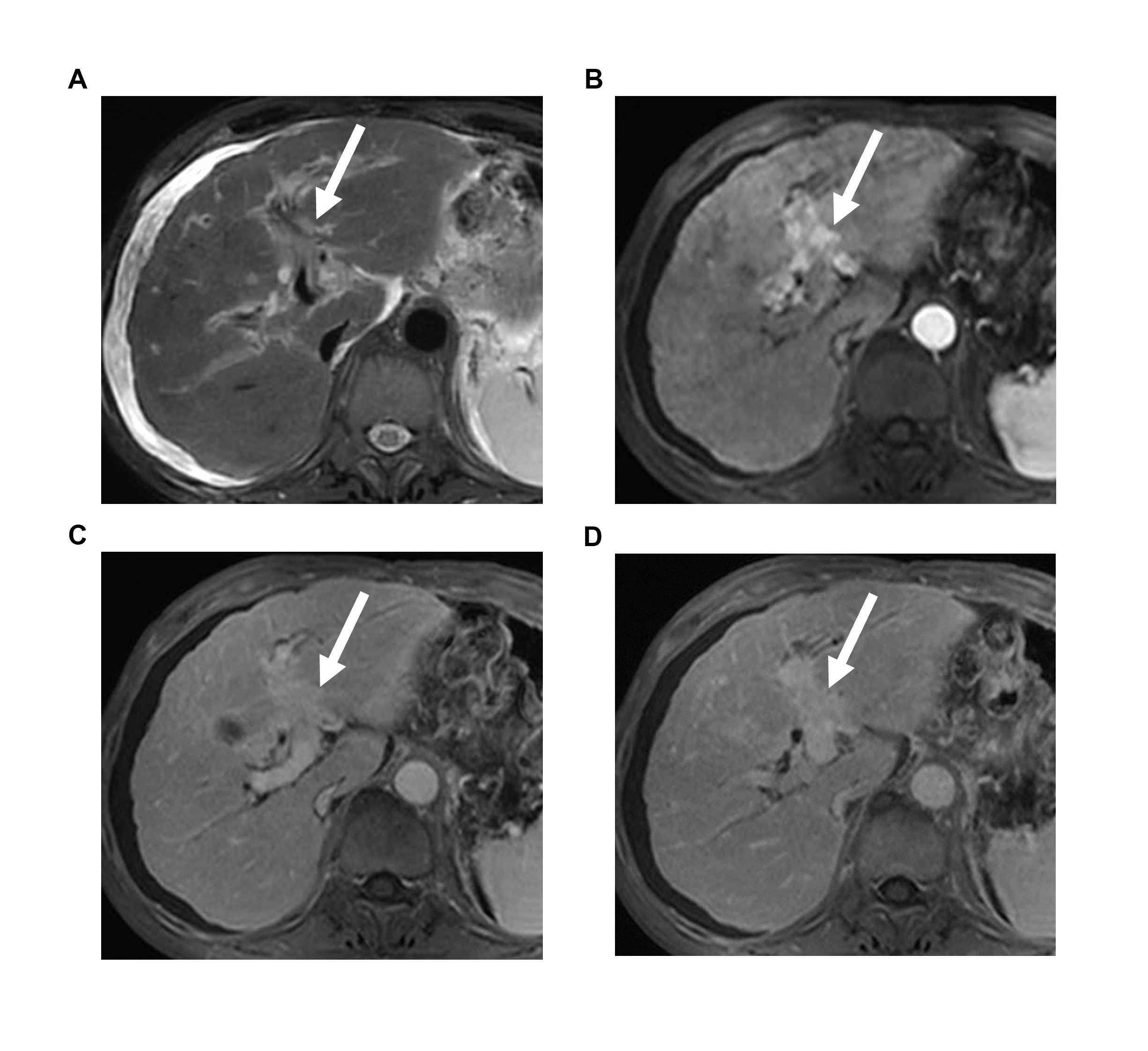

Supplement: Supplementary file 2 [file Image_2.tif]

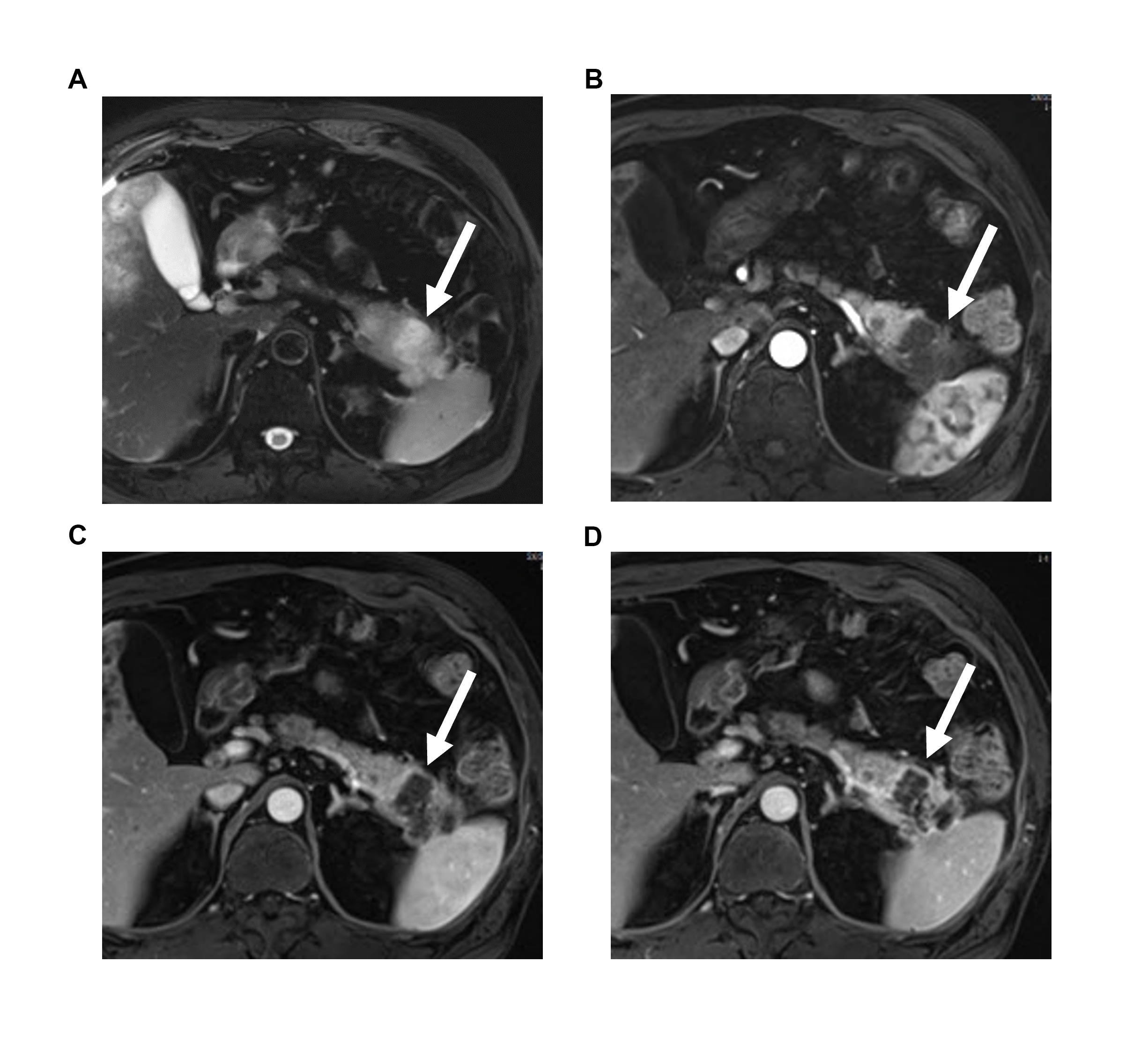

Supplement: Supplementary file 3 [file Image_3.tif]

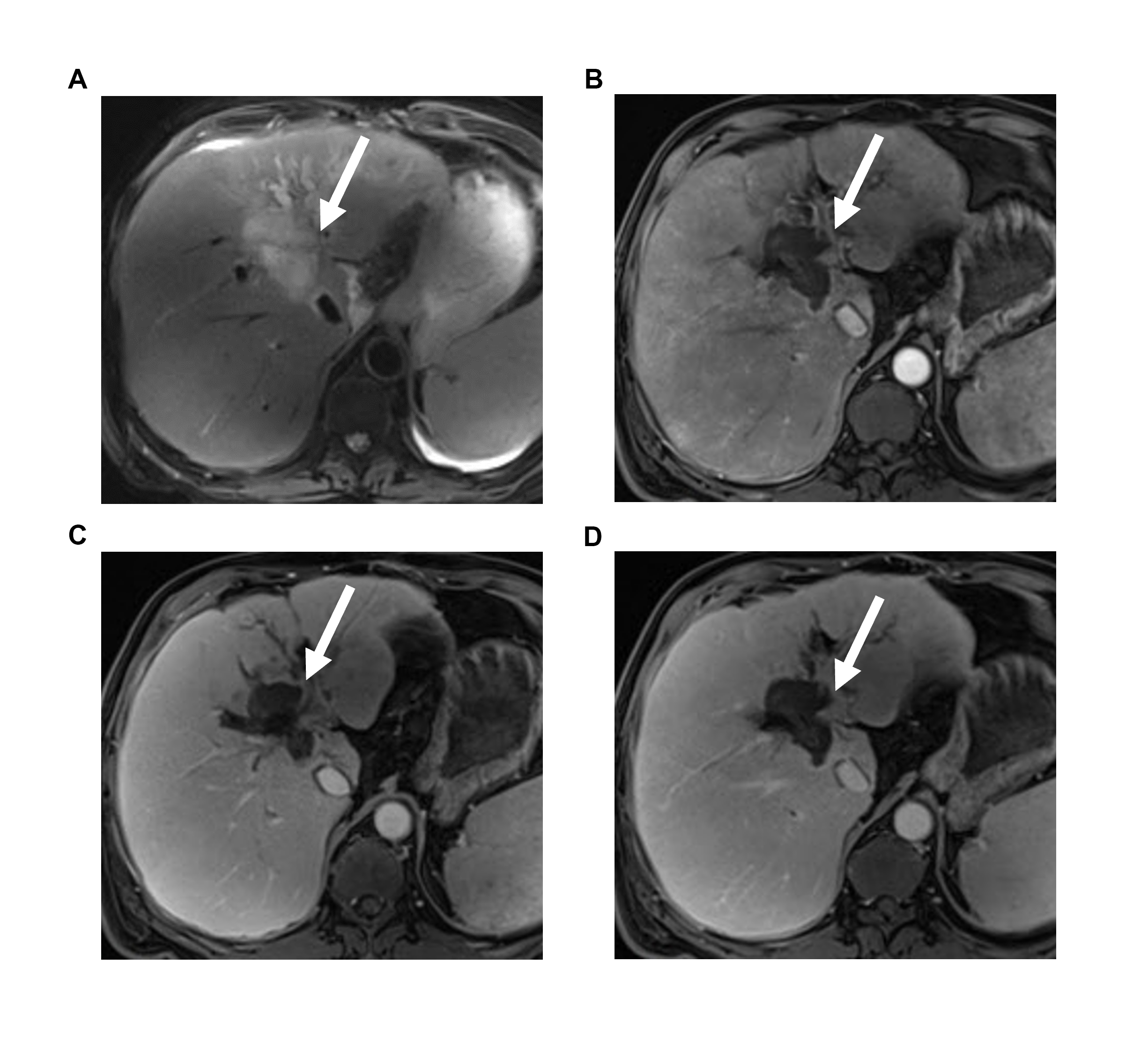

Supplement: Supplementary file 4 [file Image_4.tif]

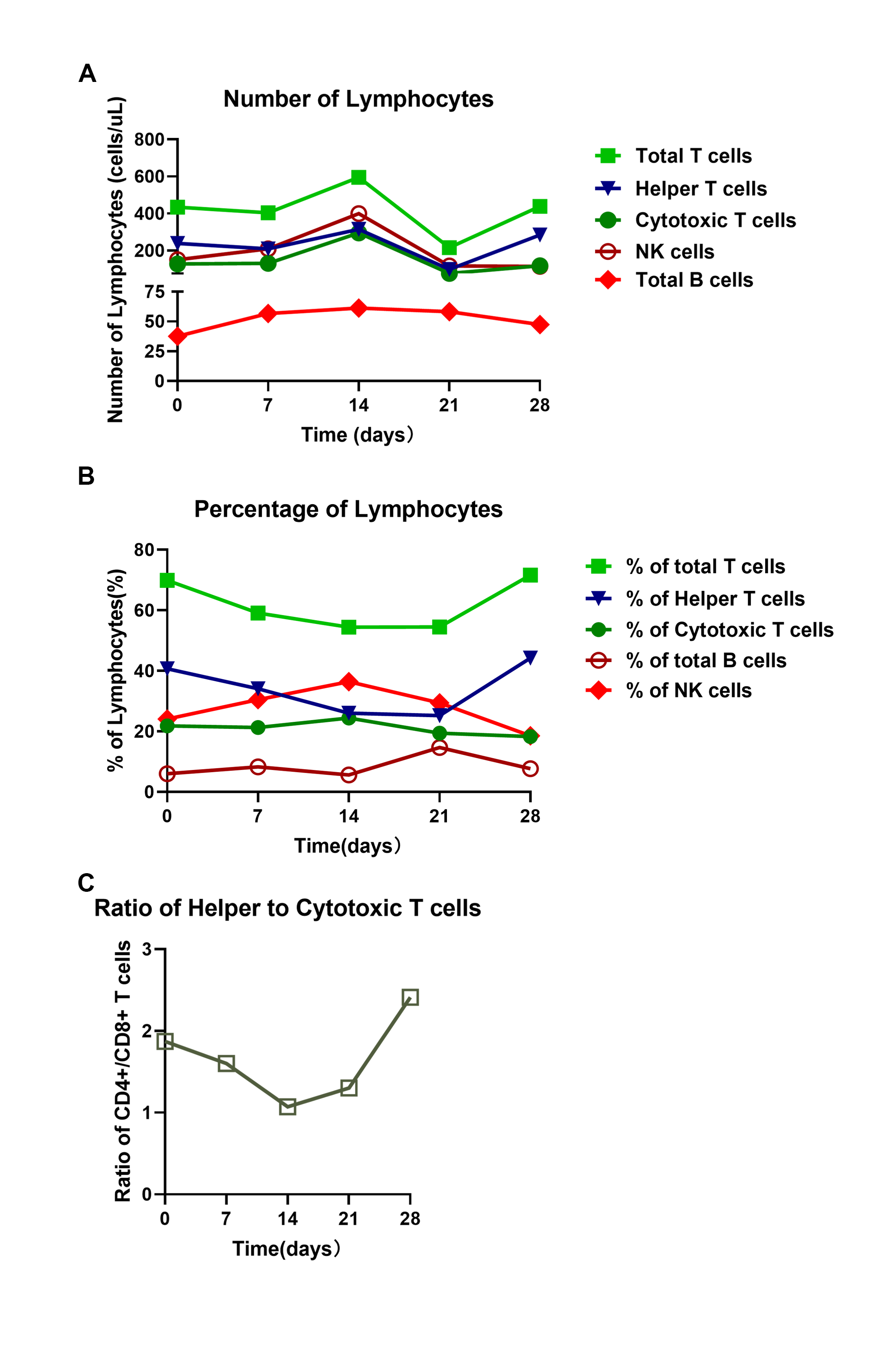

Supplement: Supplementary file 5 [file Image_5.tif]

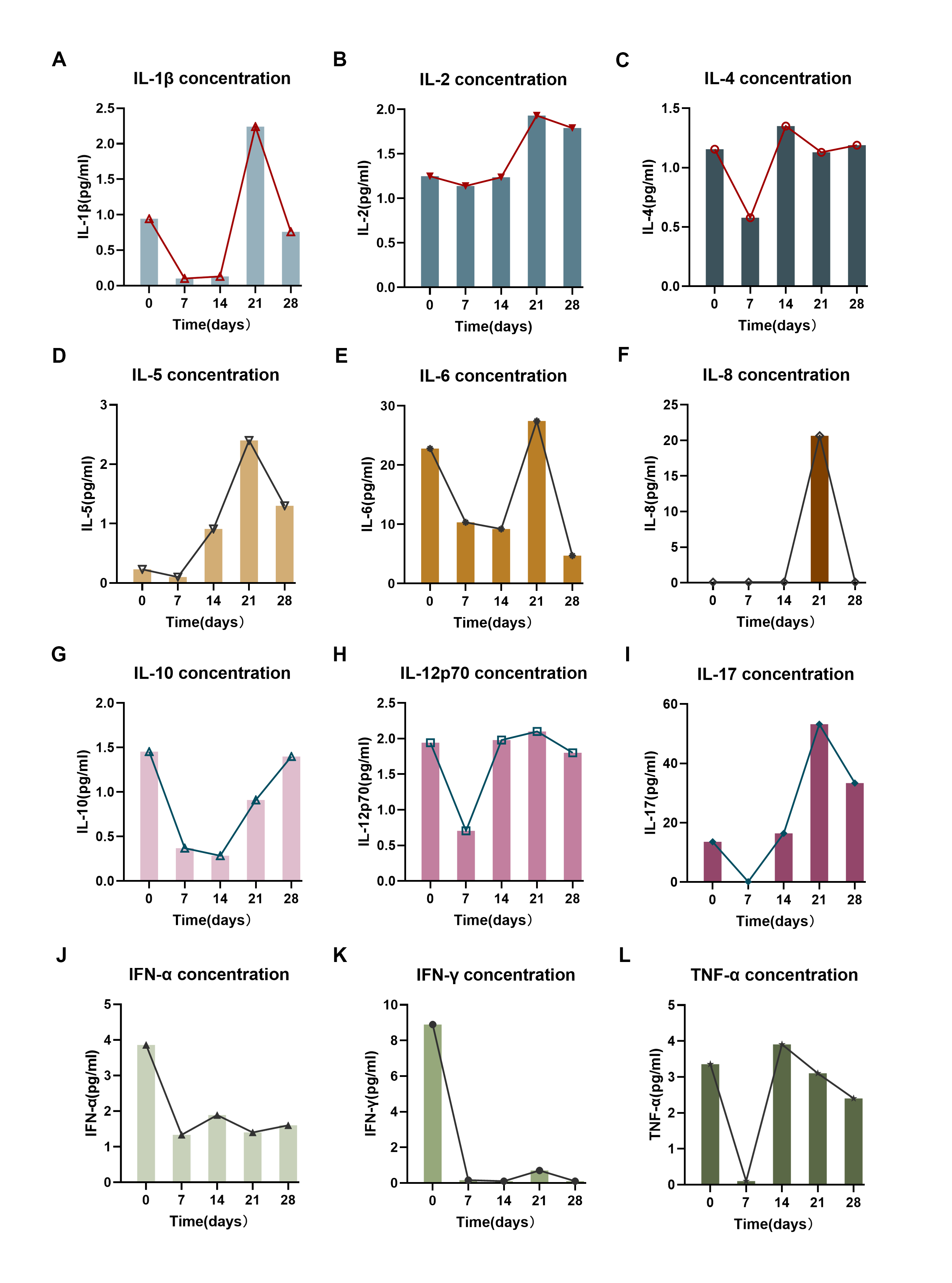

Supplement: Supplementary file 6 [file Image_6.tif]
